# Editorial: Interferons: key modulators of the immune system in cancer

**DOI:** 10.3389/fimmu.2023.1327311

**Published:** 2023-11-08

**Authors:** Nabiha Yusuf, S. Rameeza Allie, Bryan E. Strauss

**Affiliations:** ^1^ Department of Dermatology, University of Alabama at Birmingham, Birmingham, AL, United States; ^2^ Department of Microbiology and Immunology, Penn State College of Medicine, Hershey, PA, United States; ^3^ Institute of Cancer of São Paulo, Faculty of Medicine, University of São Paulo, São Paulo, Brazil

**Keywords:** interferon, immune system, cancer, immunotherapy, regulation

This Research Topic brings to the forefront the expanding knowledge on mechanisms of anti-tumor actions of interferons (IFNs) and their role in immunotherapy of cancers. IFNs comprise an extensive family of cytokines that not only possess antiviral activities, but also play a fundamental role in innate and adaptive immune regulation. There are three major types of IFNs, type I, II and III. Type I IFNs, especially IFN-α and IFN-β, can be produced by most cell types in the body in response to a wide range of stimuli and, in simplified terms, act as the initiators of many components of immune activation. In addition, they can kill tumor cells directly, sensitize cancer cells to chemotherapy and inhibit angiogenesis. Type II interferon, namely IFN-γ, produced by cytotoxic T cells and T helper cells, plays an essential role their function. Type III interferons are less well studied, but play a specific role in antiviral defense and are being considered for immunotherapy of skin cancer, including melanoma (1).

Clearly, the functions of type I and II IFNs suggest that they would be beneficial if harnessed as anticancer agents and, indeed, the interaction between IFNs, tumor cells and immune cells has been extensively studied as components of cancer immunotherapies. Even so, caution is necessary when considering IFNs, especially type I, for immunotherapy since they are also known to promote tumor growth under certain conditions, especially when low levels of IFN are maintained chronically. This may contribute to up-regulation of programmed death ligand 1 (PD-L1) on tumor cells, T cell exhaustion and the immunosuppressive tumor microenvironment (TME). In addition, systemic administration of recombinant IFN (especially α), is stymied by the short half-life of the protein, leading to the application of high doses that are known to cause toxicity. The use of IFN holds the promise of effective antitumor and pro-immunologic activity, but must be optimized to avoid the consequences of low-level chronic or high-level systemic use (2).

IFNs have been used in combination with other therapies, including cytotoxic drugs, hormones, radiotherapy and other immunostimulatory compounds. Gene therapy approaches promise localized, high-level production of IFN while avoiding the pitfalls of systemic administration. Associating immune-stimulating therapies, such as checkpoint inhibitors, with IFNs could act synergistically to decrease immunosuppression and increase the efficacy of anti-tumor response. Therapy with IFN or IFN inducers can be effective by minimizing systemic toxicity in predetermined, responsive patients.

The works that comprise this Research Topic show current progress and concepts related to the role of IFNs as modulators of the immune system and as cancer fighting agents. For example, Han et al. discuss the pathways of IFN-γ production, regulation and signaling before reviewing the stem-like properties and expression of immunosuppressive molecules by tumor cells that contribute to resistance to IFN-γ activity. The work by Chen et al. explores alterations in gene expression related to the activity of IFN-γ in a cohort of 930 pancreatic cancer patients, leading to a novel prediction model of drug responsiveness in high and low-risk groups. In this way, a prognostic model for pancreatic cancer was created based on the expression profile of IFN-γ related genes. Farhana et al. showed that gold nanoparticles act through suppressor of cytokine signaling (SOCS)1-induced nuclear factor (NF)-κB signaling to alter the expression of miRNAs in triple negative breast cancer (TNBC). While in IFN-γ stimulated cells, the application of the nanoparticles in combination with anti-miR155-5p inhibited NF-κB p65/50 activation. This suggests that the nanoparticles may be used to modulate miRNAs and thus inhibit IFN-γ induced inflammation in TNBC.

In the review by Lim et al., they explore the influence of type I IFNs on the antitumor immune response in gliomas and the immune landscape of the brain TME, pointing to the importance of type I IFN for promoting a ‘hot’, immune activating, TME. Razaghi et al. expand on this topic by focusing on the combination of type I IFN with PD-1/PD-L1 blockade, revealing increased T cell infiltration and activation, generation of memory T cells, efficacy and safety in a variety of tumor types. Even so, the need for better biomarkers to predict resistance remains a challenge. In the work by Chen et al., analyzed gene expression in ovarian carcinoma, identifying a correlation between high level expression of interferon-stimulated genes (ISGs) and improved prognosis. They then identify ISG20 as a key mediator of host-antitumor immune response since its overexpression leads to accumulation of dsRNA and increased IFN-β production. Thus, targeting ISG20 may contribute to future immunotherapies.

Martini et al. review the development of nadofaragene firadenovec, their recently approved gene therapy which utilizes a non-replicating adenoviral vector to transfer the IFN-α cDNA and its application in bladder cancer patients who did not respond to Bacillus Calmette-Guerin (BCG). This approach is intriguing since the bladder is readily accessible, the gene therapy is applied together with Syn3, an anionic detergent, to aid in gene delivery, resulting in high level expression that is long lasting and contributes to antitumor immune activation. As a result, in a phase III study, 53% of patients (CIS ± Ta/T1 BCG-unresponsive NMIBC) had a complete response at three months and 24% maintained this a year after treatment. For HGTA/T1 tumors, a 73% high grade recurrence-free survival rate was seen at three months and 44% at 12 months.

Overall, the works in this Research Topic promote the use of type I and II interferons in immunotherapy singly or in combination with checkpoint blockade. The approval of the use of various type I interferons (Roferon-A and Intron-A) in the treatment of tumors (3) is corroborated by the vital role of interferons in tumor immunity. Yet, there is limited advances in exploiting type II and III interferons in translational medicine.

As mentioned above, type II interferons may have a role in tumor regression and promotion. Further work on identifying the targets that promote tumor cell metastasis is needed to elucidate the way forward in using it in immunotherapy. As studies show that IFN-γ is also involved in the upregulation of inhibitory receptors, combining it with checkpoint blockers may be the first step in using type II interferons.

As type III interferons are restricted in signaling to epithelial tissue, they have been shown to be effective in various skin and epithelial derived tumors. Further, as they do not undergo negative feedback like type I interferons (4), they may be a more potent target for cancer vaccines.

While targeting interferon signaling is substantiated by the correlation of interferon signaling pathways with anti-tumor responses, the works in this Research Topic and others prompt us to further explore the mechanisms that drive interferon mediated resistance to other immunotherapies.

## Author contributions

NY: Conceptualization, Writing – review & editing. SA: Writing – review & editing. BS: Writing – original draft.
